# Nucleotide Sequencing and Identification of Some Wild Mushrooms

**DOI:** 10.1155/2013/403191

**Published:** 2013-12-30

**Authors:** Sudip Kumar Das, Aninda Mandal, Animesh K. Datta, Sudha Gupta, Rita Paul, Aditi Saha, Sonali Sengupta, Priyanka Kumari Dubey

**Affiliations:** ^1^Cytogenetics, Genetics and Plant Breeding Section, Department of Botany, Kalyani University, Kalyani, West Bengal 741235, India; ^2^Pteridology-Palaeobotany Section, Department of Botany, Kalyani University, Kalyani, West Bengal 741235, India; ^3^Department of Botany, Charuchandra College, Kolkata, West Bengal 700029, India; ^4^Department of Botany, Narasinha Dutt College, Howrah, West Bengal 711101, India; ^5^P.G. Department of Botany, Hooghly Mohsin College, Hooghly, West Bengal 712101, India

## Abstract

The rDNA-ITS (Ribosomal DNA Internal Transcribed Spacers) fragment of the genomic DNA of 8 wild edible mushrooms (collected from Eastern Chota Nagpur Plateau of West Bengal, India) was amplified using ITS1 (Internal Transcribed Spacers 1) and ITS2 primers and subjected to nucleotide sequence determination for identification of mushrooms as mentioned. The sequences were aligned using ClustalW software program. The aligned sequences revealed identity (homology percentage from GenBank data base) of *Amanita hemibapha* [CN (Chota Nagpur) 1, % identity 99 (JX844716.1)], *Amanita* sp. [CN 2, % identity 98 (JX844763.1)], *Astraeus hygrometricus* [CN 3, % identity 87 (FJ536664.1)], *Termitomyces* sp. [CN 4, % identity 90 (JF746992.1)], *Termitomyces* sp. [CN 5, % identity 99 (GU001667.1)], *T. microcarpus* [CN 6, % identity 82 (EF421077.1)], *Termitomyces* sp. [CN 7, % identity 76 (JF746993.1)], and *Volvariella volvacea* [CN 8, % identity 100 (JN086680.1)]. Although out of 8 mushrooms 4 could be identified up to species level, the nucleotide sequences of the rest may be relevant to further characterization. A phylogenetic tree is constructed using Neighbor-Joining method showing interrelationship between/among the mushrooms. The determined nucleotide sequences of the mushrooms may provide additional information enriching GenBank database aiding to molecular taxonomy and facilitating its domestication and characterization for human benefits.

## 1. Introduction

Mushrooms are defined as “a macro fungi with a distinctive fruiting body” [1] and are traditionally used worldwide as nutritious food and as medicinal sources [2–7] including antioxidant activity [8, 9]. Further, mushrooms are great recyclers and decomposers [10] and therefore play a significant role in the ecosystem. To date, about 3000 species are regarded as prime edible mushrooms [11]. Proper identification knowledge of edible mushrooms is essential for effective exploration in human benefits.

Molecular markers, PCR (Polymerase Chain Reaction) [12] and non-PCR based [13], are widely used for mushroom identification and characterization. However, direct sequencing of PCR product of repetitive nuclear DNA [14–16] of mushrooms is powerful tool for identification and phylogenetic studies [17]. The present study describes the nucleotide sequencing of 8 wild edible mushrooms collected from Eastern Chota Nagpur Plateau of West Bengal, India, using genomic DNA from fruit bodies. The rDNA-ITS (Ribosomal DNA Internal Transcribed Spacers) fragments of the genomic DNA were amplified using ITS1 (Internal Transcribed Spacers 1) and ITS2 primers. The nucleotide sequences of 8 mushrooms were matched from the available known sequences of GenBank database. The ITS region is rather useful for molecular characterization in fungi at the species level and within the species [11]. The objective of the work is to gain proper identification knowledge of the mushrooms, which may provide direction towards domestication and commercialization of the wild species for economic benefits apart from aiding molecular taxonomy.

## 2. Materials and Methods

### 2.1. Germplasms

Eight morphologically different types of wild edible mushrooms (CN (Chota Nagpur) [1–8]) were collected from their natural habitat in Eastern Chota Nagpur Plateau of West Bengal (Paschim Medinipur having sandy loam to loamy soil of reddish brown color cover; latitude 22°N to 25°30′N, longitude 83°47′E to 87°50′E, altitude 610 m) during the rainy (June-July) and postrainy (August to October) seasons of 3 consecutive years (2008 to 2010). The mushroom samples were characterized morphologically following the methodology suggested by Largent and Stuntz [18]. The samples verified up to genus level [19] were as follows: CN 1—*Amanita* sp.; CN 2—*Amanita* sp.; CN 3—*Astraeus* sp.; CN 4—*Termitomyces* sp.; CN 5—*Termitomyces* sp.; CN 6—*Termitomyces* sp.; CN 7—*Termitomyces* sp.; and CN 8—*Volvariella* sp.

### 2.2. DNA Isolation

Field isolated samples were dried (45°C ± 1°C) and the dried samples were used for fungal DNA isolation from fruit bodies using the Fungal Genomic DNA MiniSpin Kit (Chromous Biotech, Bangalore) as per methodology suggested by Graham et al. [20]. Isolated DNA samples were checked in agarose gel (1% agarose gel prepared in TAE (Tris-acetate-ethylenediaminetetraacetic acid) buffer to which 2 *μ*L ethidium bromide was added) to confirm the yield of DNA, purity, and concentration. A ladder (1 kb plus) was loaded in the gel in order to compare the size of the isolated DNA samples.

### 2.3. Primer Designing

Based on morphological identification of the samples, available nucleotide sequences ranging from 2 to 5 kb were downloaded from GenBank data base for primer designing. The FASTA format of all those sequences was arranged in a file and then the sequences were aligned according to their homology of nucleotide using ClustalW software program (http://www.ebi.ac.uk/Tools/msa/clustalw2/). The stretches of conserved sequences (ITS1 and ITS2) were considered for primer designing. The length of the primers was kept within 18 to 25 bp and the GC% was within 40 to 55% so that the melting temperature of the primers will not be high during PCR. It was also considered that the amplicon size should not cross more than 500 bp. Forward and reverse primers (Table 1) for 8 mushroom samples were synthesized commercially (IDT, USA) for amplification of fungal DNA.

### 2.4. PCR Reactions

Polymerase chain reaction was performed using the following: 10x PCR buffer 2.5 *μ*L, dNTPs (25 mM of each) 2.0 *μ*L, MgCl_2_ (25 mM) 2.0 *μ*L, forward primer (10 pm/*μ*L) 1.0 *μ*L, reverse primer (10 pm/*μ*L) 1.0 *μ*L, DNA template 10 ng for each sample, Taq. polymerase (2 U/*μ*L) 0.5 *μ*L, and DNase- and RNase-free water where volume was adjusted to 25 *μ*L. The PCR conditions were determined according to Taq. polymerase, primer pair, and amplicon size. The amplification reactions were performed in a DNA Thermal Cycler (Eppendorf AG, Hamburg, Germany) programed as follows: 1st cycle of 5 min at 95°C (initial denaturation) followed by 30 cycles of 45 sec at 95°C (denaturation), 30 sec at 50°C (annealing), 1 min at 72°C (extension), and 1 cycle of 10 min at 72°C (final extension). The final step was held at 4°C. The PCR products were stored at −20°C and subsequently run in agarose (Hi-media, USA; in TAE buffer) gel (1%). PCR product (10 *μ*L) was loaded in the gel by adding 6x DNA loading dye along with 2-log DNA ladder. The gel was run at 50 volts for 1 hour and the amplified products were visualized in the UV trans-illuminator and photographed in Gel Doc system (Bio-Rad, USA).

### 2.5. Sequencing

The PCR products were loaded in the coupled lane of low melting point agarose gel (Sigma, USA). The run was at 40 volts for 1 hour and the bands were cut using sterile blade. The DNA was extracted from the agarose using columns (QIAGEN, Germany). This DNA was quantified in 1% agarose gel and then sequenced using the respective forward and reverse primers. The kit which was used for DNA sequencing was Big Dye Terminator Kits, Applied Biosystems, USA and the sequencing was done in Capillary Electrophoresis DNA Sequencer, Applied Biosystems, USA.

### 2.6. Evolutionary Relationships Analysis

Following alignment of the nucleotide sequences, a phylogenetic tree was constructed using the Neighbor-Joining method [21]. The optimal tree is drawn with the sum of branch length equal to 1.4246. The tree is drawn to scale, with branch lengths in the same unit as those of the evolutionary distances used to infer the phylogenetic tree. The evolutionary distances were computed using the Maximum Composite Likelihood method [22] and are in the unit of the number of base substitutions per site. The analysis involved 8 nucleotide sequences. Codon positions included were 1st + 2nd + 3rd + noncoding. All positions containing gaps and missing data were eliminated. There were a total of 102 positions in the final data set. Evolutionary analyses were conducted in Molecular Evolutionary Genetics Analysis 5 (MEGA5) [23].

## 3. Results

**Figure 1:**
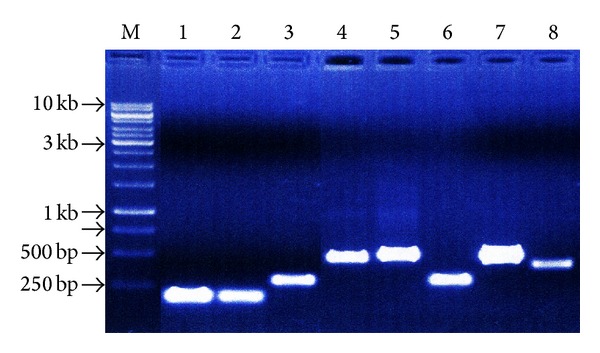
Gel purified PCR product of the field isolated mushroom samples (M marker lane, 1 kb ladder).

The size of the DNA of 8 mushroom samples was around 15 kb. The PCR amplification products (Figure 1) showed that CN 1 and 2 gave around 200 to 250 bp amplified band, while CN 3 and 6 had 300 bp amplified band. CN 4, 5, and 7 documented a band around 500 bp, while CN 8 showed 400 bp band. These PCR products were gel purified, run in 1% agarose gel, and processed for nucleotide sequencing.

**Figure 2:**
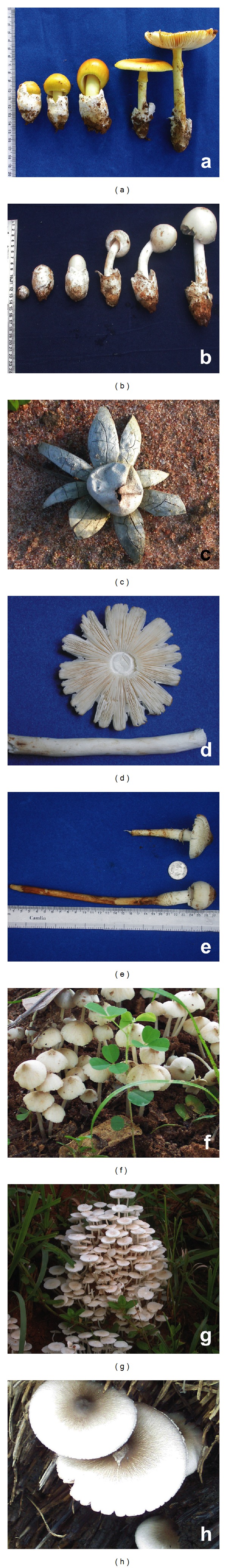
Fruit bodies of mushrooms. (a) *Amanita hemibapha*—CN 1. (b) *Amanita* sp.—CN 2. (c) *Astraeus hygrometricus*—CN 3. (d) *Termitomyces* sp.—CN 4. (e) *Termitomyces* sp.—CN 5. (f) *T. microcarpus*—CN 6. (g) *Termitomyces* sp.—CN 7. (h) *Volvariella volvacea*—CN 8.

The partial nucleotide sequences (Table 2) of 8 samples were obtained and analyzed for Basic Local Alignment Search Tool (BLAST) search program (National Center for Biotechnology Information (NCBI) site) against the whole GenBank data base of nucleotide sequences (using ClustalW software) for identification. Each PCR product was sequenced using forward and reverse primers and both were used for BLAST. The BLAST result is presented in Table 3. Based on % identity, mushrooms may be identified as follows: CN 1—*Amanita hemibapha* [family: Amanitaceae; % identity 99 (JX844716.1)]; CN 2—*Amanita* sp. [% identity 98 (JX844763.1)]; CN 3—*Astraeus hygrometricus* [Family: Diplocystaceae; % identity 87 (FJ536664.1)]; CN 4—*Termitomyces* sp. [Family: Lyophyllaceae; % identity 90 (JF746992.1)]; CN 5—*Termitomyces* sp. [% identity 99 (GU001667.1)]; CN 6—*T. microcarpus* [% identity 82 (EF421077.1)]; CN 7—*Termitomyces* sp. [% identity 76 (JF746993.1)]; and CN 8—*Volvariella volvacea* [Family: Pluteaceae; % identity 100 (JN086680.1)]. Out of 8 samples, 4 belong to *Termitomyces* spp. of which only 1 could be identified up to species level from the available GenBank data base. Molecular identification of the mushroom samples corroborated morphological identification of the samples up to genus level (Figures 2(a)–2(h)).

**Figure 3:**
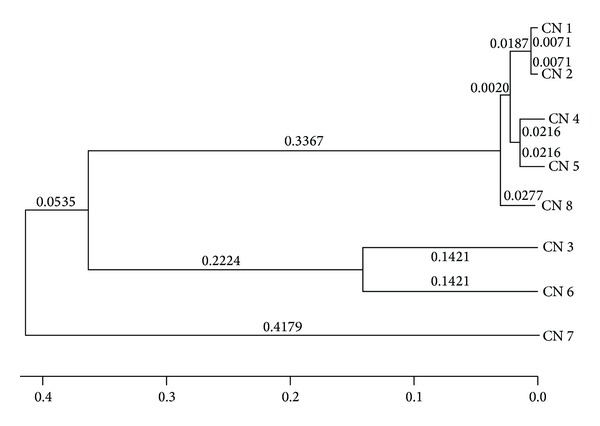
Phylogenetic tree showing relationship of mushroom samples.

Phylogenetic tree (Figure 3) constructed revealed a close relationship between CN 1 and 2 (*A. hemibapha* and *Amanita* sp.), 4 and 5 (*Termitomyces* sp.), and 3 and 6 (*Astraeus hygrometricus* and *T. microcarpus*). CN 8 (*V. volvacea*) showed close affinity with CN 1, 2, 4, and 5; however, CN 7 (*Termitomyces* sp.) showed distant relationship with other samples.

## 4. Discussion

The nucleotide sequences of 8 mushroom samples were blasted against available sequences from GenBank data base for identification. One sample, CN 8 (*Volvariella* sp.), matched 100% Indian sample* V. volvacea*, accession number JN086680.1, reported from Solan, Himachal Pradesh. On the other hand, the rest of the samples matched the samples that were reported from outside of India. However, CN 1 identified as *A. hemibapha* (pileus color: yellow) from GenBank data base matched morphologically the reported mushroom *A. hemibapha* (pileus color: white) by Roy and Samajpati [24] from Bankura district, West Bengal, adjoining to the current location of study. On the contrary, CN 2 was also identified as *Amanita* sp. that possessed white color pileus. Therefore, CN 1 and 2 could be different *Amanita* species as evinced from nucleotide sequences. CN 6 was identified to be as *T. microcarpus* from the available sequence of GenBank data base (82% homology). CN 4 and 5 belonging to *Termitomyces* spp. seem to be of genetic lineage different from that of the other *Termitomyces* spp. (CN 6 and 7) as noted from the phylogenetic tree; however, all *Termitomyces* species were collected from the same locality. From molecular point of view CN 4 and 5 were identified as *Termitomyces* spp., and these samples matched morphologically *T. clypeatus* and *T. eurhizus* [25], respectively. Tang et al. [26] also characterized *Termitomyces* species (*T. longiradicatus*, *T. quilonensis,* and *T. poonensis*) different morphologically from India. From phylogenetic tree, *V. volvacea* showed distinct relationship to *A. hemibapha* and *Amanita* sp. although the fungi belong to different families and therefore the finding is rather interesting from taxonomic point of view and may further be explored. The present study possibly suggests that GenBank data base for mushroom is not sufficiently rich in India. However, molecular characterization of 4 mushroom samples up to species level is performed and is essential and the provided nucleotide sequences of the rest of the samples may be relevant to GenBank data base for further exploration in the field.

Rajaratnam and Thiagarajan [11] extracted genomic DNA from the fruit body of wild mushroom and subjected it to nucleotide sequencing using ITS1 and ITS4 conserved primer stretches. The sequence was aligned using Jukes-Cantor Correlated Distance model and the aligned sequence (559 bp) revealed 88% matched score with *Perenniporia* sp. (GQ982890.1). Dung et al. [27] characterized 6 Oyster mushroom samples based on both morphological and molecular data and found that both were corroborating to each other. Lee et al. [16] performed identification of 3 medicinal mushroom (*Ganoderma lucidum*, *Coriolus versicolor*, and *Fomes fomentarius*) species from Korea based on nuclear large subunit rDNA sequences. Nucleotide sequencing of *Termitomyces albuminosus* [28], *Ganoderma lucidum* [29], and *Agaricus bisporus* [30], among others was also performed.

## 5. Conclusion

Molecular identification of the fungal samples may enrich and provide additional information to mushroom biodiversity and GenBank data base resource aiding to molecular phylogenetic analysis. Further, identification knowledge may also be significant for human benefits.

## Figures and Tables

**Table 1:** Primers designed for different mushroom sample amplification.

Sl. no.	Primer name	Sequence (5′ to 3′)	Size (bp)	Melting temp. (°C)	Amplicon size (bp)
1	Len-F	TGGTTACTCTACAAACACTT	20	54	
2	Len-R	CCTGCTAATGTATTTCAGAAG	21	58	331

3	Vol-F	GATCATTACAGAATCGAACGC	21	60	
4	Vol-R	CTGGGCTTGAGGATTCGATG	20	62	290

5	Rus-F	AGTGCTCTCACATACAAATATC	22	60	
6	Rus-R	GTTGAGGATGTTCACGACACTC	22	66	290

7	Ter-F	GCAACGGCACTCTATCGCTGA	21	66	
8	Ter-R	CTCCTCAGATCACCAAGGAG	20	62	321

9	Ast-F	CTCCAACTTCATCAAGAAGGT	21	60	
10	Ast-R	GATCTTGTAGACATCCTGGAGAG	23	68	230

11	Ama-F	TCTTGGCTCTCGCATCGATG	20	62	
12	Ama-R	CCAGCAATGACTCCCAATATC	21	62	200

**Table 2:** Partial nucleotide sequences of the field isolated mushroom samples.

Sample number	Sequences
CN 1	TCTTGGCTCTCGCATCGATGAAGAACGCAGCGAAATGCGATAAGTAATGTGA ATTGCAGAATTCAGTGAATCATCGAATCTTTGAACGCATCTTGCGCTCTTTGG TATTCCGAGGAGCATGCCTGTTTGAGTGTCATTAATCTCTCAAAAACACTTG GTTTTTGGATATTGGGAGTCATTGCTGG

CN 2	TCTTGGCTCTCGCATCGATGAAGAACGCAGCGAAATGCGATAAGTAATGTGA ATTGCAGAATTCAGTGAATCATCGAATCTTTGAACGCATCTTGCGCTCCTTGG CATTCCAAGGAGCATGCCTGTTTGAGTGTCATTAATATCTCAAAATACTTTAA GTGTTTTGGATATTGGGAGTCATTGCTGG

CN 3	AACTGGAGGCTCGAGGCGTCGATGGCGTCAAGGAGCGTCTTGCCCTTGACAC CGCTTTGGTTTCCTTAGTCCAGCCTTTGTACCATGGCATGCTATTCGGCGAGT CAGGTTACGTAACAGAGATGAGATTAAAACGCGCACTTAGATGATTCCTCCA ACATGTTGTCACCGTGCCATCCAGAGATGGGGACGAATGCAACAGCCTTGGG GTTGTAACCGACCTTCTTGATGAAGTTGGAGAA

CN 4	AATTTTACCTTTATCACATTTCGCTGCATTCTTCATCAATGCCTTACCAAGAA ATCTATTGCTGAAAGTTGTATTTGATTAAAGGCCTGAGGCCAATAACAAGAC ATTCTAATACATTCTTTACAAAGTGATGAAATGCATAGACCAGAAATGCGAG GAAAGCCAGTGGCAGCCCCTCAAAACTGAGAGTGTGACCCCCCAAAAGGTA TCCAAAAGTCTACAAAAGGTGCACAGATGGTTGAAAATGATGGCAGGCGTG CACATGCCCCTAGAGCCAGCAACAACCTGATCAGGTTTAATTCAATAATGAT CTTTCTGCAGGTTCACTTACAAAAACCTTGTTACTATTTTACTTCCTCTAAAT GACCAAGTTTGATCAGCTTCTCAGCGATAGAGTGCCGTTGCCTA

CN 5	TGTATTTAGAGGAGTAAAGTCGTAACAAGGTTTCCGTAGGTGAACCTGCGGA AGGATCATTATTGAATTTAAACCCTGGTTGGGTTGTTGCTGGCCTCTAGGGG CATGTGCACGCCTGCCACCGTTTTCAACCACCTGTGCACCTTTTGTAGACTTT GGATATATACCGTTCGAGGGTCAAACCCCCTCCTCGGTTTTGAGGGCTTGCT GTGCTGCAAAGTTCGGCTTCCCTTGCATTCCCAGTCTATGCATCTTCCTATAC CCCGTAATGAATGTATTAGAATGTTTTTTTATTGGCCTTTTTAGTGCCTTTAAT CAAATACAACTTTCAGCAACGGATCTCTTGGCTCTCGCATCGATGAAGAACG CAGCGAAATGCGATAAGTAATGTGAATTGCAGAATTCAGTGAATCATCGAAT CTTTGAACGCACCTTGCGCTCCTTGGAGAACAGAAGGAGAAGA

CN 6	TCGTACCCAAAGAAGGGATCGATCATACCATATTTTTAACAATTTTTGATGG TTATTTTCAGCATGCATGGTACAAGGGTGGACCAAGGAGACCAAGGCTAGTG TTTCAAGGCAAGACCCTTCACAAACCTTCATCCTTGACCCCCTTTCTCCATCA AAAAAACTTAAAAG

CN 7	ACCCTCCCCCAAACCAGGGTTTGGACCCCCCGGCCCGGATCCAAAACGGGG GTTAAAAACAAGGGCGGGGGCCCTTGCCTCTAAAGGCCAACACCCCACCCG GGTTTAATTCAAAAAGGACCCTTCCCAGGTTCCCCTACGAAAACCTTGTTCA ACTTTTATTTCCCCAAAAACCAATTTTAACCTTTCCCCCAATAAAGGCCCTTT ACCAA

CN 8	GATCATTACAGAATCGAACGCGGGTCGGGCTGATTGCTGGCTCCTCGGAGCA GGTGCACGCCCTCCCCGACGCCTTCCATTCTCCACGTCCCCACCTGTGCACCT TCTGTAGGCCGTGAAGCCGCCTCGTTCGGCTCCCTCGGCTCTACGAGATCTTT TGTACACCCTTGAGAAAAACGTGTTGCAGAGTGTTCTTGTACGACCGGGGAC CCCTCGTCGGCCCCATAGACATACCAATACAACTTTCAACAACGGATCTCTT GGCTCTCGCATCGATGAAGAACGCAGCGAAATGCGATAAGTAATGTGAATT GCAGAATTCAGTGAATCATCGAATCTTTGAACGCACCTTGCGCTCTTAGTAA TGTGAATTGCAGAATTCAGTGAATCATCGAATCTTTGAACGCACCTTGCGCT CTT

**Table 3:** Summary of the BLAST result.

Samples	Sequence length blasted (bp)	Highest coverage	% identity (accession no.)	Identified name of samples
CN 1	185	100%	99 (JX844716.1)	*Amanita hemibapha *
CN 2	187	100%	98 (JX844763.1)	*Amanita* sp.
CN 3	250	95%	87 (FJ536664.1)	*Astraeus hygrometricus *
CN 4	368	99%	90 (JF746992.1)	*Termitomyces* sp.
CN 5	463	93%	99 (GU001667.1)	*Termitomyces* sp.
CN 6	161	67%	82 (EF421077.1)	*Termitomyces microcarpus *
CN 7	177	90%	76 (JF746993.1)	*Termitomyces* sp.
CN 8	359	100%	100 (JN086680.1)	*Volvariella volvacea *
